# A Cold Case: Cold Agglutinin Disease Complicating Stem Cell Transplant

**DOI:** 10.1155/crit/6270387

**Published:** 2026-02-24

**Authors:** Majed W. Al-Nazer, Sara Alsammerai, Hemalatha G. Rangarajan, Rolla F. Abu-Arja

**Affiliations:** ^1^ Pediatric Blood and Marrow Transplant Program, Nationwide Children′s Hospital and The Ohio State University, Columbus, Ohio, USA; ^2^ Ohio University Heritage College of Osteopathic Medicine, Columbus, Ohio, USA, ohio.edu

## Abstract

Allogeneic hematopoietic cell transplants (HCTs) offer a curative option for patients with life‐threatening hematologic diseases. While stem cell infusion reactions are common, they are generally not severe and typically do not affect engraftment. We present a rare case of isolated secondary cold agglutinin syndrome (CAS) in a donor, complicating the graft infusion and causing poor graft function (PGF) in a recipient. The 27‐year‐old recipient, diagnosed with refractory T‐cell acute lymphoblastic leukemia (ALL), underwent a 9/10 HLA‐mismatched unrelated peripheral blood stem cell transplant. The donor developed an upper respiratory infection 1 week before the donation, and cold agglutinin antibodies were detected in the donor′s stem cell product. During infusion, the patient developed fever, tachycardia, and hypertension. Despite initial neutrophil engraftment and full donor chimerism, the patient remained transfusion‐dependent, indicating PGF. A subsequent CD34‐selected stem cell boost from the same donor on Day +108 posttransplant, confirmed to be CAS‐negative, resulted in successful platelet engraftment and recovery. This case highlights the potential complications of CAS in donors, emphasizing the need for comprehensive donor evaluation and management strategies to mitigate adverse effects on recipients.

## 1. Introduction

Stem cell grafts obtained from both related and unrelated donors give a curative option to many patients with life‐threatening hematologic diseases requiring allogeneic hematopoietic cell transplants (HCTs). Although adverse events during stem cell product infusion are common, they are generally not life‐threatening and mostly do not affect engraftment [1]. Herein, we present the first known case of isolated secondary cold agglutinin syndrome (CAS) in the donor, complicating the graft infusion and likely causing poor graft function (PGF) in a stem cell transplant recipient. This case is unique in its hypothesis that cold agglutinin activity in the donor product resulted in potential CD34+ stem cell clumping, reducing the effective cell dose delivered to the recipient and leading to PGF. To our knowledge, this mechanism has not been reported in the literature and adds a new dimension to evaluating postinfusion complications. Relevant mechanisms of cold agglutinin impact and immune‐mediated graft dysfunction are discussed in this context [2, 3].

## 2. Case Report

A 27‐year‐old male with spastic cerebral palsy and ileocolic Crohn′s disease was diagnosed with refractory T‐cell acute lymphoblastic leukemia (ALL) and was referred to Nationwide Children′s Hospital in January 2024. He underwent chemotherapy according to the Children′s Oncology Group study AALL0434 and achieved morphologic remission (with minimal residual disease <1%). One month after induction, he presented with blurry vision and was found to have leukemic cells in the retina, followed by frank bone marrow relapse. He received orbital radiation followed by two cycles of chemotherapy (Cycle 1: fludarabine and Ara‐C; Cycle 2: nelarabine, etoposide, cyclophosphamide, PEGylated asparaginase, and dexamethasone), achieving a second remission.

The patient (blood type O+, CMV+) proceeded to a 9/10 HLA‐mismatched unrelated peripheral blood stem cell (PBSC) transplant using a 30‐year‐old male donor (blood group A+, CMV+). He received total body irradiation (1200 cGy) and cyclophosphamide (120 mg/kg)‐based myeloablative conditioning, along with posttransplant cyclophosphamide, tacrolimus, and abatacept for graft‐versus‐host disease (GVHD) prophylaxis.

The donor PBSCs were collected on Day −1 and Day 0. There was no evidence of red cell agglutination at donation, and no PBSC collection issues were encountered for the donor.

Both products arrived fresh at our cell therapy lab for processing. On receipt, the bag showed clotting and sedimentation (Figure 1a). Microscopically, the product showed the presence of cold agglutinin antibodies (Figure 1c). Through the National Marrow Donor Program (NMDP), the donor had recently experienced a respiratory infection treated with azithromycin. Symptoms resolved by collection, and both the collection center and NMDP providers reviewed these details and were comfortable proceeding with the donation as planned.

Figure 1Effects of cold agglutinins on the allogeneic PBSC product. (a) Photograph of the peripheral blood stem cell (PBSC) collection bag on arrival at the cell therapy laboratory. The unmanipulated product contains a mixture of donor stem cells and red blood cells. At room temperature, cold agglutinins in the donor′s plasma caused visible macroscopic clots and sedimentation of RBCs within the bag. (b) Appearance of the same PBSC product after gradual warming to 37°C before infusion. The clots seen in (a) have dissipated, and the supernatant is uniformly turbid, illustrating that rewarming reversed RBC agglutination. The product was administered via a blood‐warming device to prevent reactivation of cold agglutinins during infusion. (c) Microscopic view of an aliquot of the PBSC product stained with trypan blue. Numerous red blood cells aggregated into small clusters or rouleaux, which are typical of antibody‐mediated agglutination that persists even when macroscopic clots have dissolved.

**Figure:**
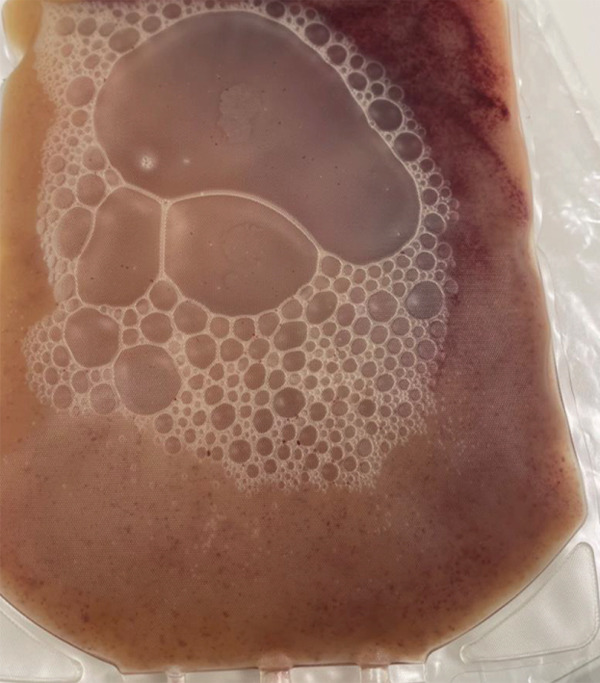
(a)

**Figure:**
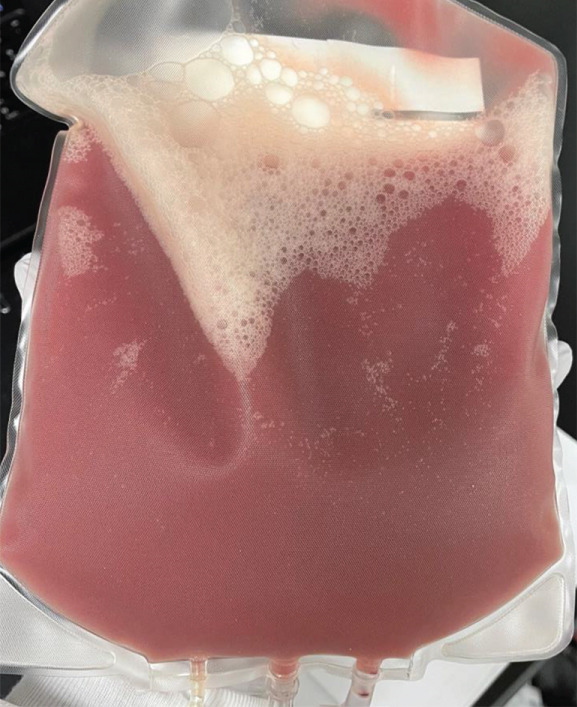
(b)

**Figure:**
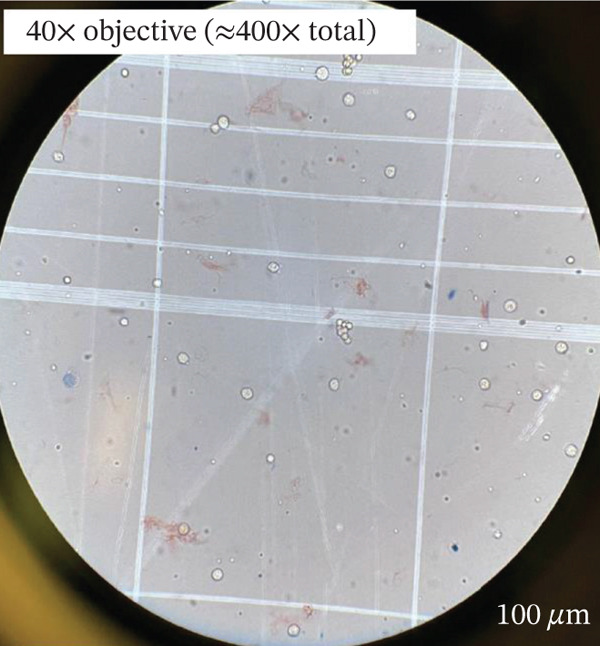
(c)

Given that the patient had already received myeloablative conditioning and a backup donor was not immediately available, a decision was made to infuse the product. Therefore, our patient received the unprocessed, allogenic PBSC infusion (Bag 1 contained CD34 9.54 × 10^6^/*k* *g*). Bag 2, containing CD34 6 × 10^6^/*k* *g*, was cryopreserved. The infusion was done via a central line and was run through a blood warmer before the infusion. The macroscopic clotting resolved by warming the bag, and the entire product from Bag 1 was infused over 6 h.

Approximately 1 h through the infusion, the patient developed a fever (max: 40.0°C) and rigors and was noted to have tachycardia (up to 180 bpm) and hypertension. Hemolysis labs were drawn and showed a mildly elevated LDH (482 U/L, normal: 140–280 U/L), normal haptoglobin (169 mg/dL, normal: 30–200 mg/dL), normal plasma hemoglobin (30 mg/dL, normal: < 50 mg/dL), and normal indirect bilirubin (0.4 mg/dL, normal: 0.2–1.2 mg/dL). The direct Coombs test was negative. Complete blood count (CBC) revealed anemia (Hb: 5.8 g/dL; normal: 13–17 g/dL), leukopenia (WBC: <0.1 × 10^3^/*μ* *L*), and thrombocytopenia (platelets: 23 × 10^3^/*μ* *L*; normal: 150–400 × 10^3^/*μ* *L* *s*). Blood cultures drawn during the episode were negative, and he was started empirically on broad‐spectrum antibiotics. He also received meperidine, methylprednisolone, acetaminophen, and diphenhydramine for treatment of possible transfusion‐related reactions. Overnight, the patient remained febrile, and further management with 1 g/kg of intravenous immunoglobulin (IVIG) was initiated. The fever resolved the following morning, and his clinical status stabilized.

He engrafted neutrophils on D +22 but remained transfusion‐dependent and needed intermittent use of growth factors (both granulocyte colony–stimulating factor and thrombopoietin agonists). His posttransplant course was complicated by GVHD (GI Stage II, Grade III), *Clostridium difficile* infection, *Klebsiella pneumonia*, BK viremia, acute kidney injury, and veno‐occlusive disease (VOD). His VOD was managed with defibrotide and continuous renal replacement therapy. Peripheral blood chimerism at D +30 and D +60 posttransplant showed 98%–99% donor chimerism. All his transplant complications resolved except for persistent cytopenia, which was suggestive of PGF in the setting of full donor chimerism. Bone marrow on Day +70 showed marked hematopoietic hypoplasia with cellularity less than 10%, with no evidence of leukemia. Given his PGF, he received a CD34‐selected unconditioned stem cell boost (dose 5.01 × 10^6^/*k* *g*) from the same donor on Day +108 post‐HCT. The donor was evaluated and tested for cold agglutinin before collection and was confirmed to be negative at this collection. The patient engrafted platelets on Day +13 of the stem cell boost, and he remains 100% donor chimerism, transfusion‐independent with count recovery, without GVHD, and in remission at 6 months posttransplant (Table 1).

**Table 1:** Patient timeline from transplant day to last follow‐up.

Day (relative to transplant)	Event
Day 0	9/10 HLA‐mismatched PBSC transplant (CD34 9.54 × 10^6^/*k* *g*)
Day +22	Neutrophil engraftment
Day +30	Donor chimerism ~98%–99%, transfusion‐dependent
Day +70	Hypocellular marrow (< 10% cellularity)
Day +100	Persistent cytopenia (poor graft function)
Day +108	CD34‐selected stem cell boost (5.01 × 10^6^/*k* *g*)
Day +13 from boost	Platelet engraftment & transfusion independence
~6 months	Remission & transfusion independence, no GVHD

## 3. Discussion

Cold agglutinin disease is a rare autoimmune disorder characterized by cold agglutinin antibodies that bind to red blood cells at subnormal body temperatures, causing agglutination and hemolysis [2]. CAS, in contrast to primary disease, occurs secondary to infections or malignancies and is typically transient. Most cases are associated with *Mycoplasma pneumoniae* but may also be triggered by Epstein–Barr virus, cytomegalovirus, and SARS‐CoV‐2 [3]. This report is significant as it highlights how transient donor CAS can affect stem cell product integrity and recipient outcomes. Cold agglutinins can be either clinically significant or insignificant. Clinically insignificant cold agglutinins may cause only in vitro agglutination without systemic symptoms. In our case, the donor exhibited evidence of cold agglutinins but lacked clinical features of CAS, suggesting a transient, in vitro nuisance phenomenon.

PGF is defined as cytopenia and transfusion/growth factor dependence despite full donor chimerism and absence of relapse or drug/infection‐related causes [4, 5]. Known risk factors include low CD34 cell dose [6]. Our patient received a robust dose (CD34 9.54 × 10^6^/*k* *g*) yet experienced PGF. We hypothesize that CD34+ cells clumped due to donor CAS, effectively reducing the viable cell dose delivered. The infusion reaction (fever, hypertension, and tachycardia) also supports an immune‐mediated etiology.

The donor′s recent upper respiratory infection likely caused the CAS, leading to agglutinins in the donor′s product. The product was kept warm throughout the infusion to prevent it from sticking to the lines. This bag was also intermittently massaged to ensure flow. Despite these measures, the infusion rate was slow. Our patient also developed an infusion reaction, as evidenced by developing high fever, tachycardia, and hypertension resolved following the administration of supportive medications.

Major ABO mismatch (recipient O, donor A) may have compounded these effects. ABO mismatch is known to delay red cell engraftment and can rarely contribute to pure red cell aplasia (PRCA). Although our patient had no overt hemolysis or PRCA, we acknowledge the potential additive risk. IVIG was administered empirically to prevent autoimmune hemolysis. Although its role in CAS or ABO mismatch is unclear, it may have blunted further immune activation.

This case highlights two major challenges: infusion complications and posttransplant cytopenia in the absence of obvious hematologic relapse or infection. Cold agglutinin antibodies, while classically associated with hemolytic anemia, have not been described as a direct cause of complications in HCT settings. This adds to the limited literature on donor‐derived immunologic interference with graft quality and expands awareness of nontraditional risks in stem cell transplantation.

## 4. Conclusion

Donor CAS, even when secondary and transient, can contribute to infusion reactions and potentially poor graft outcomes. In urgent transplant settings, where the donor product must be used, clinicians should anticipate complications and prepare accordingly. Warming products, premedicating recipients, and careful monitoring are essential. This case underscores the need for awareness and further study of donor‐derived factors impacting transplant success.

## Author Contributions

Majed Wail Al‐Nazer and Sara Alsammerai contributed equally as first authors.

## Ethics Statement

Informed consent was obtained from the family.

## Conflicts of Interest

The authors declare no conflicts of interest.

## Data Availability

The data that support the findings of this study are available from the corresponding author upon reasonable request.

## References

[1] Vidula N. , Villa M. , Helenowski I. B. , Merchant M. , Jovanovic B. D. , Meagher R. , Mehta J. , Singhal S. , Winter J. N. , Frankfurt O. , Altman J. K. , Williams S. F. , and Gordon L. I. , Adverse Events During Hematopoietic Stem Cell Infusion: Analysis of the Infusion Product, Clinical Lymphoma Myeloma and Leukemia. (2015) 15, no. 11, e157–e162, 10.1016/j.clml.2015.08.085.Epub, 26482109. 26482109

[2] Berentsen S. , How I Treat Cold Agglutinin Disease, Blood. (2021) 137, no. 10, 1295–1303, 10.1182/blood.2019003809, 33512410. 33512410

[3] Berentsen S. , Cold-Antibody Autoimmune Hemolytic Anemia: Its Association With Neoplastic Disease and Impact on Therapy, Current Oncology Reports. (2024) 26, no. 9, 1085–1096, 10.1007/s11912-024-01569-8, 38874820. 38874820

[4] Carreras E. , Dufour C. , Mohty M. , and Kroger N. , The EBMT Handbook: Hematopoietic Stem Cell Transplantation and Cellular Therapies, 2019, 7th edition, Springer, https://www.ncbi.nlm.nih.gov/books/NBK553978/. 32091673

[5] Sureda A. , Carpenter P. A. , Bacigalupo A. , Bhatt V. R. , de la Fuente J. , Ho A. , Kean L. , Lee J. W. , Sánchez-Ortega I. , Savani B. N. , Schetelig J. , Stadtmauer E. A. , Takahashi Y. , Atsuta Y. , Koreth J. , Kröger N. , Ljungman P. , Okamoto S. , Popat U. , Soiffer R. , Stefanski H. E. , and Kharfan-Dabajas M. A. , Harmonizing Definitions for Hematopoietic Recovery, Graft Rejection, Graft Failure, Poor Graft Function, and Donor Chimerism in Allogeneic Hematopoietic Cell Transplantation: A Report on Behalf of the EBMT, ASTCT, CIBMTR, and APBMT, Bone Marrow Transplant. (2024) 59, no. 6, 832–837, 10.1038/s41409-024-02251-0, 38443706. 38443706 PMC11161398

[6] Halahleh K. , Arai Y. , and Gavriilaki E. , Post-Transplant Complications, Blood Cell Therapy. (2023) 6, no. 1, 23–29, 10.31547/bct-2022-021, 37324567. 37324567 PMC10266915

